# Patients' perceptions of their experiences with nurse-patient communication in oncology settings: A focused ethnographic study

**DOI:** 10.1371/journal.pone.0199183

**Published:** 2018-06-18

**Authors:** Engle Angela Chan, Fiona Wong, Man Yin Cheung, Winsome Lam

**Affiliations:** 1 School of Nursing, Faculty of Health and Social Sciences, The Hong Kong Polytechnic University, Hong Kong; 2 School of Optometry, Faculty of Health and Social Sciences, The Hong Kong Polytechnic University, Hong Kong; Hamad Medical Corporation, UNITED STATES

## Abstract

**Background:**

The nursing shortage and its impact on patient care are well-documented global issues. Patients living with cancer as a chronic illness have many psychosocial problems and often lack adequate support as a result of ineffective nurse-patient communication. A review of the literature on factors influencing the delivery of psychosocial care to cancer patients indicates that the delivery of psychosocial care in routine cancer nursing within a biomedical healthcare system has not been widely explored.

**Objective:**

To explore patients’ perceptions of their experiences with nurse-patient communication in an oncological clinical environment.

**Method:**

A focused ethnographic study was undertaken in two oncology wards of a hospital in Hong Kong. Data were collected through observations of the ward environment, the activities and instances of nurse-patient communication, semi-structured interviews with patients, and a review of nursing documents.

**Results:**

Two main themes were identified: 1. Nurses’ workload and the environment and 2. Nurse-patient partnership and role expectations. Within these two themes were related subthemes on: Sympathy for the busy nurses; Prioritizing calls to the nurses; Partnership through relationship; Nurses’ role in psychosocial care; and Reduction of psychosocial concerns through physical care.

**Conclusions:**

Many cancer patients do not expect to receive psychosocial care in the form of emotional talks or counseling from busy nurses, but appreciate the attention paid by nurses to their physiological and physical needs. Nurse-patient partnerships in cancer care may reduce the potential workload of nurses. The psychosocial needs of cancer patients could be optimized by providing good physical care through effective communication within a time-constrained oncology setting.

## Introduction

Nurses’ communication with cancer patients is a recognized challenge and an ongoing issue, where the focus has been on the holding of difficult conversations [1]. Given the frequent contact that nurses have with patients, nurses are expected to assume this important role. The substantial need that cancer patients have for information and emotional support through effective communication is well documented [2]. While numerous studies on nurse-patient communication, primarily of a quantitative design, have focused on its effectiveness in relation to psychosocial care, a recent study [3] found that oncology inpatients gave not only doctors but also nurses a lower rating on their provision of information than on their technical and interpersonal skills. There is also a limited amount of evidence on the effect on patients of an environment characterized by a lack of time. Chan et al.’s work [4] uncovered nurses’ use of routines to talk to patients given the time constraints, and Thorne et al.’s [5] findings noted the untoward effects of time pressures on patients’ perceptions of time mismanagement and on effective communication. The findings from conventional studies have been applied to communication training at the basic and continuing professional development levels, but improvements have been incremental at best [6]. This is largely because of an exclusive emphasis on the perspective of the healthcare provider rather than on that of patients [7]. Given the mutual nature of communication, studies are needed that focus more than on the patients’ share of the conversation than has hitherto been the case [8]. Thorne’s [9] findings on patients’ perceptions of patterns of poor communication with healthcare providers indicated that variations remain across contexts, cultures, and conditions. Ultimately, the paradigm shift towards patient-centeredness places demands on us to understand the experiential reality of the communication of patients with nurses that is essential to providing holistic cancer care, particularly in an Asian setting [10].

### Background

Given early detection and advances in medical treatment, many cancer patients have been living with their disease for many years, receiving long-term curative or palliative cancer care [11]. Across their cancer trajectory, they commonly experience both physical symptoms and distress resulting from the cancer itself or from the anti-cancer treatment [12]. However, the findings reveal that nearly 50% of newly diagnosed cancer patients and those with recurrent cancer do not receive adequate psychosocial support, and show a significant level of distress [13]. There are also an estimated 26 million of new cancer cases per year worldwide, which calls for psychosocial care and support to be the sixth vital sign in the standard routine of cancer nursing care [14]. Despite much evidence from around the world of the need to improve psychosocial care for cancer patients, gaps and barriers to its delivery still exist [15]. The barriers include the unwillingness of patients to share their concerns, the inability of nurses to pick up cues from patients or handle patients’ emotions, and a focus on the part of nurses on completing tasks [10], with time constraints being an important external factor. Busy nurses who do not have much time for patients apart from carrying out their routines are a global and well-documented phenomenon [16]. Time pressures, which are closely tied to heavy workloads and understaffing, would hinder patients from disclosing their concerns and expressing negative emotions to healthcare professionals [17]. Time pressures also restrain nurses from supporting patients emotionally through effective communication [18]. It may not be possible to create more time within the growing complexity of a very busy cancer care environment [19]. A lack of discussion and effective communication between nurses and patients on psychosocial issues is another predominant cause of inadequate psychosocial care [20–21].

#### Factors influencing effective communication

Andersen and Risør [22] have argued for the importance of contextualization and how it relates to the notion of causality for eventual clinical usefulness. A study on patients with malignant lymphoma identified the domains of patients’ attributes, healthcare professionals’ attributes, and external factors as barriers to effective communication [23]. Another study identified characteristics of patients, nurses, and the environment as general influences on communication [24]. Patient attributes, including negative emotions, a lack of specific knowledge about their disease, and inadequate communication skills could undermine the confidence of patients in communicating with healthcare professionals [25]. Some patients found it challenging to know and remember what to ask. Jotting down their questions on a piece of paper became their means of communication. When patients regarded their physician as a higher authority who played an important role in their cure, they were more inclined to follow instructions without asking questions. Patients’ past negative experiences, such as the feeling of not having been cared for, could also hinder their subsequent communication with healthcare professionals [23]. It would be easier for patients to raise questions or concerns if they were familiar with their healthcare providers and had developed rapport with them [26–27]. Moreover, their perceptions of the emotions or manners of the nurses could influence the building of rapport, which in turn could affect their readiness to express their feelings and needs [27–28]. Hence, while reviews have been conducted of studies on the factors that influence communication with cancer patients, there appear to have been no studies on the delivery of psychosocial care in routine nursing practice involving the collecting of qualitative data from patients [19]. Arthur Kleinman’s [29] explanatory model provides us with insights on what is most important to patients, and presents the notions of illness, culture, care, and the healthcare system as concepts, rather than entities. Notwithstanding the biopsychosocial emphasis in our healthcare system, the human experience of illness is often disregarded. A patient’s explanatory model and views of clinical reality can be quite different from the professional medical model. By freeing ourselves from ethnocentric and medicocentric views, healthcare providers may become more aware of important issues that have been systematically ignored in the clinical reality. In addition, it is not known whether the factors influencing communication that have been identified in studies conducted in the West can be applied to the situation in Hong Kong, or how Hong Kong patients’ perceptions of communication are influenced by the Chinese culture.

The number of new cancer cases and cancer deaths has been increasing in recent years [30], and is predicted to continue to rise as the population ages [31], yet cancer services are barely sufficient to deal with the current cancer burden. As cancer treatment is costly, 90% of cancer patients are treated in public hospitals [32]. However, only 6 out of the 42 public hospitals in Hong Kong provide clinical oncology services. The nurse-patient ratio in a ward is around 1:11 in Hong Kong, whereas the international standard is 1:4–6 [33]. Nursing shortages as well as the overwhelming responsibilities shouldered by nurses are the challenges that need to be addressed to improve the quality of the care offered in Hong Kong oncology settings [32, 34].

### The study

The aim of this study was to explore patients’ perceptions of their experiences with nurse-patient communication and the psychosocial care delivered within a time-constrained oncological clinical environment. The findings may reduce the gap between the rhetoric on the provision of psychosocial care for oncology patients and the meaning of such care to the patients.

## Materials and methods

### Design

A focused ethnographic approach was adopted since such an approach is suitable for investigating specific beliefs and practices of particular healthcare processes as held by patients and practitioners [35] and for focusing on cultures and subcultures framed within a particular context [36]. In this study, we explore patients’ perspectives of their nurse-patient communication, as a continuous problem, identified in a distinct subculture of cancer nursing care within a busy biomedical context [36]. Data collection and analysis were performed based on the principles of the ethnographic approach, which highlights the need to pay close attention to a distinct issue in cultures or subcultures in a specific setting, and to observe, describe, and understand how people’s behavior is influenced by the culture in which they live. This is done by immersing researchers in the culture, and by endeavoring to ensure that they enter the study without preconceptions [37]. Research commenced after ethical approval was obtained from the Kowloon West Cluster Research Ethics Committees of the hospital and the Departmental Research Committee of Hong Kong Polytechnic University.

### Sample

The study site was an oncology unit comprised of two 32–36 bed oncology wards in a hospital. Participant recruitment was completed through convenient, purposive sampling and a snowballing technique. Upon recruitment, information sheets were handed out with a verbal explanation of the study. During patient recruitment, two nurse research assistants worked with the attending nurses, who helped to identify potential patient participants. Once the patients were identified, the nurse research assistants approached them directly, explained the study, and asked them if they were willing to participate. The patient participants were all local people. The criteria for the inclusion of patients in the study were as follows: patients who were at least 18 years of age, able to communicate in Cantonese, cognitively functioning, and in reasonable enough health to be interviewed during their hospitalization. Patients with tracheotomy or who were receiving palliative or hospice care were excluded. Initially, 102 patients were recruited, but nine of them withdrew, leaving 93 patients. There were various reasons for the withdrawal. Six were discharged without having completed any procedure; one person’s condition deteriorated and the patient passed away; another was not fit enough to be interviewed after participating in a procedure and was also subsequently discharged, and yet another was later found to be unsuitable for the study. In the end, there were 47 female and 46 male participants, for a relatively gender-balanced sample.

The criteria for the inclusion of nurses were those with at least one year of nursing experience and one year in the current work setting. This ensured that they had a working knowledge of the culture of the unit. The initial total number of nurses recruited was 26. Two nurses withdrew, with one resigning and the other refusing to continue due to the busyness of the ward. In the end, 24 nurses were recruited.

All of the participants gave their written informed consent to take part in the study and provided their demographic data. They were assigned an individual code, which was used throughout this paper to maintain their anonymity.

### Data collection

Two nurse research assistants assumed the role of observers as participants in the ward environment. Observations took place over a seven-month period from March to October 2016. They were part of the research team, who also conducted focused observations of the practices of nurses, the activities of the general nurses, and instances of nurse-patient communication within the oncology settings. They both were experienced nurses with degrees, who had previously worked in cancer wards. One had a master’s degree. They discussed their observations to obtain a more complete and detailed picture of the oncology settings [38]. This overcame the problems of observer reliability and bias [37]. The participants also became accustomed to the presence of the researchers, thereby minimizing the Hawthorne effect [39]. Despite their past research experience, they were guided through their first few field visits with the principal investigator and the team members, and trained in the process of collecting data. The team started off holding regular weekly meetings, and then monthly meetings, for the research assistants to report on their data collection process and observations. The observations, which were initially recorded in a small booklet as field notes, were reported to the principal investigator and discussed with the members of the research team to generate a contextualized understanding of the culture in the ward. An expanded account of the observations was written on the basis of these notes.

Before and after each admission, administration of medication (AOM), and wound-dressing procedure, the patient participants were asked to complete a checklist of any physical and/or psychosocial concerns they might have [40]. Each participant was given a verbal explanation of how the study was going to be conducted prior to each procedural observation. Audio-taped data were collected of the nurse-patient verbal communication that occurred during these routine procedures. After the care procedure, a semi-structured interview with the patient participants, which lasted approximately 10–25 minutes, was conducted and audio-recorded. An interview guide was used (S1 and S2 Files). The interview started with the following introductory question: Were you able to express your needs in general? Why or why not? Other open-ended questions were then used as a guide to elicit the views of the patients, such as on whether they perceived nurse-patient communication to be important; what perceived facilitators and barriers affected their communication with nurses, and why. Many patients shared their thoughts in depth with examples; a few completed the interviews, but their responses were shorter due to fatigue. The collected data were summarized with the patients to give them a chance to make further comments or corrections/clarifications on the spot. Data were deemed to be saturated when the researchers noted redundancy in the data [41]. However, data continued to be collected from two more patients to ensure that no new themes would arise from the consecutive interviews. The audio-taped conversations were transcribed verbatim by experienced student helpers. The student helpers were given a briefing with a template on notation preferences, in particular, on inaudible sections and conversations with emotional contents. One of the nurse research assistants also checked the completed transcriptions.

### Data analysis

Our analysis was guided by Hammersley and Atkinson’s “grounded theorizing,” where, while there is no particular script for analyzing ethnographical data, it is essential that “data are materials to think with” [42] and not only to be managed. The interview transcripts and field notes were regarded as units of analysis. They were read and compared for contextual understanding. The research nurse assistant (the second last author) and I (the first author) created the open coding independently through reading and re-reading the interview transcripts using key words, phrases, sentences, and paragraphs, reducing the data to codes. Differences in coding were resolved through discussions. Codes were constantly compared [43] for their conceptual similarity or related meanings and for their differences by going back to the original text. They were then grouped into subcategories/subthemes. Revision of codes was an iterative process, including the backward and forward data assessment and analysis leading to modification and verification of the categories/themes. This was followed by an axial coding [43] with the intent to clarify how the emergent subcategories was related to the preliminary categories, and the relevant codes were further discussed and conceptualized. Regular data sessions [44] were conducted, in which the other two authors involved with the first and the second last authors to review the coding, mutually agreed on the codes, and reached a consensus on how to apply the created codes to the data [45]. This led to broader perspectives and caused us to move beyond preconceived beliefs and biases in collaborative reflexivity [46]. Notwithstanding the value of coding for similarities and differences, it was important to immerse ourselves in the data by repeatedly reading the text until insights were developed [47].

Agreement on the major themes was reached through extensive iterative discussions among the authors. Areas of consistency across the subthemes were sought to confirm the major themes that provided the best description of the culture being studied [48]. There was also the triangulation of data from field notes written during ethnographic observations, transcribed interviews of nurse-patient communication during procedural care, interviews with patient participants, and a document review. These strategies provide a better understanding of the culture being studied [49] and also serve the purpose of validation. The transcripts that were made of the nurse-patient interactions and interviews provided a particular view of the individual in the culture of a very busy oncology unit, validating the meanings and interpretations of the rich points observed during the fieldwork.

## Results

Two main themes were identified: 1. Nurses’ workload and the environment and 2. Nurse-patient partnership and role expectations. Illustrative quotes from the interviews and notes from the field observations are included below. Different patients have been coded using numbers; PN denotes patients in the North Ward and PS patients in the South Ward.

### Nurses’ workload and the environment

The heavy workload of the nurses was evident in the shortage of nurses and the time constraints faced by the nurses, who were engaged in many nursing routines and documentation procedures. The patients recognized that the nurses were busy, which influenced their views of the nurses’ roles and their interactions with the nurses. The crowded and noisy physical setting of the wards also did not facilitate nurse-patient communication. This theme consists of two subthemes: 1. Sympathy for the busy nurses; and 2. Prioritizing calls to the nurses.

#### Sympathy for the busy nurses

As many cancer patients had been admitted frequently and had experienced long admission times, they were able to develop a good understanding of the nurses’ work demands, which made them feel sympathetic towards the nurses. The following quotation from a patient is illustrative.

There is too much paper work, but then you wouldn’t have any records if you don’t have the paper work. Nurses are really too busy. Every day, the shift handover is done by a quarter after three in the afternoon and then non-stop till nine without any supper. I can see that the nurses in this cancer ward have worked very hard … administering medications, taking temperatures, changing diapers, weighing the patients, doing x-rays. They are simply so busy that they wouldn’t have time to talk to patients.” PN38

The various demands on the nurses were also observed and recorded in the field notes, which illustrated how the nurses undertook their work in a busy ward, and how their work patterns and their need to move quickly and multi-task would hinder nurse-patient communication.

*The nurses are at their busiest during the doctor’s round on weekdays*. *… They rush in and out of the cubicles many times*. *They also run from one patient to another in the ward*. *Commonly*, *they are providing procedural care for one patient while replying loudly to another neighboring patient in the cubicle*. (Field notes)

Apart from the nurses’ workload, the physical setting of the ward was found to influence nurse-patient communication as well. The crowded ward environment, the background noises, and the distance between the patients’ rooms and the nursing station were not conducive to communication.

*One cubicle is crammed with ten or more beds*. *It is common to see a crowded environment full of camp beds and stretchers in the corridor*. *The distance between the patients is so close that their conversations with nurses are easily overheard by their neighbors*. *It is likely that the patients’ privacy is being breached when needs or concerns are disclosed*. *The patients’ coughs*, *cries*, *and moans*, *along with the sounds emitted from vital sign monitors*, *electrocardiogram (ECG) devices*, *and intravenous (IV) infusion pumps*, *make the environment too noisy for communication*. *Isolation rooms are less crowded and quieter*. *However*, *the location is far away from the nursing station*. *The nurses only approach the patients during routine care and when the patients ask for help*. *The patients there have even less contact with the nurses*. (Field notes)

In essence, the patients understood that the nurses had a heavy workload because they had observed the nurses’ routines, fast movements and engagement in multi-tasking. The crowed and noisy physical environment provided us with more background for understanding that, for patients, the act of talking to the nurse was not an easy one, as it would require some planning on the parts of the patients. The lack of privacy and noise levels in the ward could discourage patients from engaging in any private conversations with the nurses. This, and the patients’ sympathetic understanding of the nurses’ work demands had an impact on the decisions that the patients made and the priorities that they set in getting help. The patients were essentially trying to be supportive of the nurses’ work patterns in their making of requests and sharing concerns.

#### Prioritizing calls to nurses

The patients understood that the nurses were too busy to fulfill all of their needs given their busy work within time constraints. Thus, they would only bring up concerns that they perceived to be urgent–mainly those concerning their physiological changes and needs.

“Yesterday, when I seemed to have high blood pressure, as it was as high as 190/108 in the afternoon, I asked the nurse to take it [my blood pressure] again at night-time. I think it is a serious problem that I need to raise immediately….” PS41

Patients would also look for an opportune time to raise concerns, which were primarily of non-physical nature, which they perceived to be less urgent. They were considerate of the nurses in that they tried not to interrupt them.

“I wonder why I can’t do better with the [colostomy] bag, despite practicing everyday…. [I’ll] just ask [the nurses] when they are free to come in.” PN25

Patients learnt to make their requests, so as to receive timely responses from busy nurses. The patients’ “effective” communication with the nurses was also promoted through the building of relationship. According to Gordon [50], relationships do not exist solely on their own; rather, they are developed between nurses and patients through constant physical, medical, and technical encounters, with all three types of encounters being intricately connected. With the building of a relationship, a partnership could develop between the patients and nurses.

### Nurse-patient partnership and role expectations

In any partnership, the people who are involved will assume roles–in this case, the roles of nurse and patient. They will work towards a common goal. This theme has three subthemes, 1. Partnership through relationship; 2. Nurses’ role in psychosocial care; and 3. Reduction of psychosocial concerns through physical care.

#### Partnership through relationship

A sense of partnership was observed to have developed between the nurses and the patients as they built a relationship. A relationship could be established through the transfer of information as a goal in an instrumental biomedical conversation [51], with the involvement and participation of the cancer patient. Even if such participation is minimal, it provides a basis for the development of comfort, confidence, and trust. The following dialogues were extracted from the nurse-patient interactions in an AOM procedure, they show how the development of a partnership was facilitated between the nurse and the patient during routine care.

When the nurse was administering chemotherapy, she gave the patient a pertinent explanation and information, and reminded the patient to report any adverse reactions, saying *“because chemotherapy … is a big deal*.*”* The patient replied, “*[I] now know … [I] must ring the bell for you if [I have problems during chemotherapy]*. *[I] depend entirely on you*, *not on any of the others*,*”* expressing the sentiment that he regarded the nurse as the most trustworthy person to offer him help when he needed it. *“Yes*, *you’re right*. *[We] also depend on you … [we] really need [your help]*,*”* was the nurse’s response, which gave the patient the encouragement and confidence to communicate his needs. Such a conversation reflected their interdependent and collaborative/supportive relationship, which seemed to show that they could rely on each other to contribute to the patient’s care and health. With the establishment of rapport and the patient’s familiarity with his own condition because of the long duration of his disease and his frequent admissions to the hospital, the patient could also partner with the nurses and share some of the responsibility of caring for himself by expressing his needs.

However, relationship building process could be hindered by the patients’ negative experiences arising from misunderstanding. One patient described how a misunderstanding about a request relating to her diet made her less ready to communicate with nurses when she was in doubt about the medication administered by the nurse, which then led to further misunderstandings and a difficult relationship.

“I don’t know when I’ll be discharged. If I knew I was going to leave today or tomorrow, I wouldn’t ask [the nurse] to change the meal. Since I thought I wouldn’t go home so soon, I asked the nurse to change the meal for me, but she said ‘sometimes … congee and sometimes … rice, change again and again.’ I sensed that I was troublesome to her and she didn’t like me … this made me upset. I dare not communicate and talk anymore with her in the future.” PS27

Patients’ negative experience of their interactions with nurses would inevitably shape their subsequent communication with them. Patients would be less motivated to disclose their feelings and needs to nurses. Apart from the importance of developing a relationship and partnership with the nurses, patients’ perceptions and expectations of the roles of nurses also influenced how willing the patients were to express their psychosocial needs.

#### Nurses’ role in psychosocial care

Given the patients’ past experiences with the nurses’ emphasis on physical care, the patients’ lack of familiarity with the responsibilities of nurses and their perception of the ability of nurses to provide psychosocial care led the patients to hold few expectations of nurses in this area.

“… [I] haven’t talked about my worries … [the nurses] can’t solve the problems; actually, they can’t help because they have their own responsibilities. They have already done a lot for [the patients]. If they were social workers or chaplains, then I would talk [about my concerns] because they would be specialists in counseling, that is, in helping [patients] gain relief.” PN35

Given the high cost of chemotherapy medications, financial issues were often a stressor for cancer patients. However even if patients experienced financial difficulties, they seemed to prefer to approach a doctor instead of a nurse, as illustrated below.

“I haven’t mentioned [my financial concerns] to the nurses here. They think I don’t need target therapy at the moment, then [I] don’t need to ask anymore. I’ll just leave it to the doctor to talk about [my financial concerns] when I really need [target therapy].” PS34

Patients did not often expect to receive psychosocial care from nurses. Indeed, the physical aspect of cancer patient’s condition is the prime concern of nurses, especially in time-pressured environment.

#### Reduction of psychosocial concerns through physical care

Nurses placed a higher priority on delivering physical care when time was tight and they could not expect to meet the patients’ psychosocial needs. However, the patients expressed much appreciation for the help that they received from the nurses in fulfilling their physical needs. Despite the lack of focus on psychosocial care, the patients’ psychosocial comfort could also be, and was, enhanced through the provision of good physical care during procedures.

“At least I feel that [nurses] can help me…. As nurses have their own professional role, they are not [there] to take care of our psychological needs…. I’ve already felt ‘psychologically better’ when they are in their professional role of administering medications. Just like before, I told the nurse that I had a headache. She asked me ‘Do you need any analgesics to relieve the headache?’ Actually, this is what she has already done in her profession. She has given me suggestions on how to solve the problem. At least she can help me relieve my headache. That’s already enough.” PS45

Besides alleviating the physical pain of the patients, which gave the patients psychological comfort, the nurse would provide the patients with an explanation of their physical condition, which could also ease the patients’ fears.

“The nurse explained to me why my legs are weak…. [Her] assessment, information, and reassurance about my physical needs mitigated my worries.” PS27

Repeated hospitalizations gave cancer patients the opportunity to observe the heavy workloads of the nurses. However, the building of a relationship between the nurses and the patients meant that the two parties interacted continuously, underscoring the potential for forming a partnership in care, which not only could help the patients adhere to their care regime, but also perhaps alleviate some of a nurse’s workload. Conversely, patients’ negative experience of their interactions with nurses could cause the patients to lose the motivation to disclose their emotional concerns to nurses, although, admittedly, many of the patients in this study were not aware of the role played by nurses in providing psychosocial care. The few that seemed to recognize that nurses could play this role, perceived that the time constraints on nurses made it impossible to receive psychosocial attention from them. This suggests that while the provision of psychosocial care to patients might not be something that is expected or even possible given the patients’ understanding of the role of nurses and the time constraints, one way of reducing the psychosocial concerns of patients is for both nurses and patients to focus on the physical needs of patients as the priority.

## Discussion

### Communication in the context of time pressures

This study shows that patients often did not explicitly express their needs because of time pressures, which is an organizational barrier to communication [23]. It is well recognized that understaffing is the main cause of time pressures [52]. Previous studies have also shown that the influence of Chinese culture can inhibit patients from disclosing their needs. Patients feel embarrassed about bothering the nurses, so that they express their physical pain only when it has become intolerable [21]. In our study, the patients’ patterns of communication with the nurses also seemed to be shaped by their understanding of how busy the nurses were and by the pattern of the nurses’ routines. Whether or not the patients decided to initiate communication depended on whether they thought that their problem should be promptly solved or could be further delayed until the nurses came to provide procedural care. If the patients believed that their problem was life-threatening or intolerable–usually when they experienced physiological changes or physical pain–they would seek immediate help from the nurses, regardless of how busy they thought the nurses were. Previous studies have indicated that patients seldom engage in active discussions with nurses on psychosocial problems such as those arising out of worries about their finances. Chinese patients are more reserved than Westerners about openly discussing sensitive topics with healthcare professionals [53]. They are ashamed to receive help from a social worker, and will not talk about their financial problems [54]. In the present study, the patients also rarely brought up the subject of their finances. However, rather than relating to Chinese cultural influences or to feelings of embarrassment, the reason that they did not discuss the problem of finances seem to relate more to their perception that the financing of their treatment was a secondary concern that did not need to be mentioned or urgently resolved–or which would become urgent only when the need arose. Coupling the importance of being involved in their own care with an understanding of how busy nurses are with their work, patients would prioritize their needs and delay reporting them, or not report needs that they perceived to be less urgent. This, however, could pose safety issues.

In addition, healthcare professionals can be reluctant to become involved with hospitalized cancer patients in fear of placing a burden on a vulnerable group [55]. This attitude, and the adoption by nurses of the role of being the experts, could cause nurses to have doubts about the ability and motivation of the patients to participate in their care.

### Building rapport for partnership and communication

The concept of partnership was central to the patients’ self-control and ownership of the management of their symptoms [25]. The findings [3] revealed that hospitalized patients who were more actively involved in their own care often initiated conversations and approached nurses for information pertaining to their illness and self-care. The effective exchange of information is critical to the ability of clinical nurses to assess and educate cancer patients and their families, perform symptom management, and coordinate care. This, in turn, promotes nurse-patient communication and facilitates nurse-patient partnerships. As in other studies, this study found that nurses are skilled at eliciting clinical information to empower patients and at building therapeutic relationships [56]. Being empowered, patients could become more proactive at engaging in self-care activities [57]. Similarly, in the current study, some “experienced” patients were found to be helpful at lightening the workload of the nurses, since they understood how busy the nurses were and readily shared some self-care responsibilities. Patient empowerment was found to be a feasible way to promote physical self-care through a nurse-patient partnership in a time-constrained oncology setting.

The Hospital Authority [34] also advocates the forming of nurse-patient partnerships through effective communication as a key strategic direction in improving the quality of care in Hong Kong. Empowering patients to care for themselves is considered to be the optimal approach to managing physical conditions. Currently, this approach is being applied to some cancer out-patients through the launching of self-management courses [58]. Patient involvement in physical self-care can also be put into practice within in-patient oncology settings, as the present study shows that patients could be empowered with the ability to monitor themselves and report on their own condition during chemotherapy. Since the success of a partnership requires interpersonal and communication skills from healthcare professionals [59], the patient’s perception of a negative attitude on the part of the nurses can probably be attributed to inadequate communication skills and a lack of mindfulness on the part of the nurses, who are used to taking a factual approach when speaking to patients without being aware of the impact that their words could have on the patients [60]. Baillie [59] acknowledges that personal reflections on working experiences and feedback from colleagues are of value to the professional development of nurses.

### Awareness of the role of nurses in cancer care

This study found that there was a widespread belief among patients that nurses have a major role to play in managing symptoms, but little to do with providing psychosocial care. Many others were unaware of the role played by nurses in psychosocial care and were reluctant to express their psychosocial concerns [54]. Nurses could play many different roles in psychosocial care, for instance, by assessing needs, acknowledging distress, managing symptoms of distress, clarifying treatment options, educating the patient about variations in distress during the transition period, building trust, clarifying access to resources, and providing assistance with referrals for emotional needs such as counseling [12]. However, our findings show that many oncology patients did not regard the provision of psychosocial care, such as acknowledging emotional distress and engaging in counseling, as the kinds of roles undertaken by nurses. The few who did think that nurses could have such roles wondered whether they were close enough to the nurses to discuss their psychosocial concerns with them. Patients have been observed to prefer to seek emotional support from family members and friends rather than from healthcare professionals [61].

It could also be asked whether all cancer patients require emotional support, since previous studies have revealed a significant variance in the need for such support depending on the type of malignancy suffered by the patient. Patients with leukemia and lymphoma were more likely to report having had discussions about emotional issues (58.1%) than those with thyroid cancer (17.4%). The age and race of the patient are also factors that influence the need of the patient to discuss the psychosocial implications of his/her cancer. Further investigation on the subject is needed to provide insights to guide psychosocial care planning for patients [62].

### Close connection between physical and psychosocial needs

This study may heighten nurses’ awareness of the interactions between physical and psychosocial needs and the related aspects of care for their patients in a time-critical environment. The interrelationship between physical needs and psychosocial management was demonstrated in the present study, which showed that the psychological state of the patients changed with the physical care that they received. The art of managing symptoms has been defined as the skill of coping with the distress arising from experiencing symptoms [63]. Studies have elaborated on how profoundly symptom management can affect the psychosocial aspects of a patient’s life. Unrelieved physical symptoms, such as pain, fatigue, sleeplessness, vomiting, and constipation, are considered risk factors for distress [13]. In this study, good physical management involving effective nurse-patient communication in procedural care promoted psychosocial comfort in the patients. In this busy ward, the general focus on cancer care was for nurses to provide physical care to patients, treating the psychosocial aspect as something separate. Surprisingly, the patients also accorded a lower priority to their psychosocial needs, and had no expectations that the nurses would play a psychosocial role. This finding complements, yet differs from, Dilworth et al.’s finding [19] that the barrier to receiving psychosocial care most frequently reported by patients (38.77%) is their view that there is “no need for psychosocial services and support,” followed by a lack of information and not knowing that the service is available. Similar to the findings of [63,64], the present study shows that nurses frequently considered their core task to be medical management, and that both nurses and patients placed an emphasis on the physical care provided by the nurses. This suggests that a focus on the physical comfort of the patients could enhance the patients’ psychological well-being. Therefore, nurses could consider promoting psychosocial comfort in patients by improving the quality of their physical care through effective communication during nursing procedures and when providing symptom relief. This is a possible solution to achieving the nursing goal of balanced care within the available timeframe.

Following through with Kleinman’s explanatory model for our findings on the concepts of illness, culture, and care has reminded us that despite the emphasis on the biopsychosocial model, particularly for cancer care, nurses and patients continue to live in a culture of biomedical dominance. It is important for nurses and other healthcare providers to take into account patients’ concepts of psychosocial care in working with busy nurses, and their perception of the importance of nurse-patient communication. The findings provide an alternative view of patients’ appreciation of the importance of the physical care delivered by nurses for their psychosocial health, so that there is a need to re-examine the dualistic view of mind and body, and to integrate the findings into practice.

## Conclusion

Patients’ perceptions of the busyness of the nurses and the clinical environment will alter their patterns of communication. They appreciate the demands on nurses, but could be persuaded to communicate more openly if they build a relationship with the nurses. They could be empowered to partner with the nurses to become involved in their care. However, negative perceptions or misunderstandings of the attitude of the nurses could affect the patients’ desire to communicate with the nurses and the feasibility of forging a partnership with them.

This study also implies that there is a need to improve the nurse-patient relationship by encouraging nurses to strengthen the practice of mindfulness and improve their communication skills. In addition, the patients’ limited disclosure of their psychosocial concerns relates not only to their perception of their relationship with the nurses, but also to their perception of the roles played by nurses and of the nurses’ competence in providing psychosocial care. Another key finding of this study is the interrelationship between psychosocial care and the physical needs of patients. While physical and psychosocial care could be considered separate matters for cancer patients, the provision of good physical care through effective communication is the key to promoting the psychosocial well-being of patients. This may be the optimal way to realize the goal of providing holistic care to cancer patients within Hong Kong’s understaffed oncology settings.

### Implications for practice

From the findings, the following recommendations for practice can be made, which may improve nurse-patient communication from the perspective of the patients.

First, given that cancer patients will prioritize their needs before considering whether or not to ask nurses for help, it is important for nurses to empower those patients, yet work closely with them, by continuously assessing and monitoring changes in the patients’ condition, only then will patients have the ongoing ability to act as partners with busy nurses in managing their own care.

Second, considering patients’ perceptions of the importance of rapport, it is essential for nurses to become more aware of their communication skills and personal attitudes. It may be useful of holding informal and flexible reflective workshops for nurses to learn about communication through a model of appreciation rather than deficit. The support of hospitals is paramount for nurses to be able to reconstruct the clinical reality of their communications and dialogue with peers.

Third, since patients did not consider, or were unaware of the role of nurses in providing psychosocial care, particularly emotional counseling, it would be helpful for nurses to listen to and understand the views of patients in order to bridge the gap between the patients’ expectations and the actual role of nurses.

Lastly, the patients’ appreciation of the impact of physical care on their psychosocial needs offers busy cancer nurses, who would only be able to attend to the psychosocial needs of patients when time permits, an alternative way of attending to such needs.

## Supporting information

S1 File Semi-structure interview guided questions (English). (DOCX)

S2 File Semi-structure interview guided questions (Chinese). (DOC)
