# Supplementary material for: Patients' perceptions of their experiences with nurse-patient communication in oncology settings: A focused ethnographic study
Source: PLoS One. 2018 Jun 18;13(6):e0199183. doi: 10.1371/journal.pone.0199183 (PMC6005521; doi:10.1371/journal.pone.0199183)
Supplement: S1 File — (DOCX) [file pone.0199183.s001.docx]

**S1 File**

Semi-structure interview guided questions.

1. Are you able to express your needs in general? If so, why? If not, why not?

2. Do you think it is important to tell nurses about your needs? If so, why? If not, why not?

3. In your experience, how did you tell the nurses about your needs and concerns?

4. How do nurses respond to your needs and concerns? Are you satisfied with their responses?

5. What were the reasons for you to express your needs and concerns to nurses?

6. What were the reasons for you not to express your needs and concerns to nurses?
