# Supplementary material for: Patients' perceptions of their experiences with nurse-patient communication in oncology settings: A focused ethnographic study
Source: PLoS One. 2018 Jun 18;13(6):e0199183. doi: 10.1371/journal.pone.0199183 (PMC6005521; doi:10.1371/journal.pone.0199183)
Supplement: S2 File — (DOC) [file pone.0199183.s002.doc]

**S2 File (original Chinese language)**

**訪問病人問題**

1. 在一般情況下，你會否說出自己的需要? 為甚麼?
2. 你認為把你的需要告訴護士是否重要? 為甚麼?
3. 根據過往經驗, 你是怎樣和護士提出你的需要和擔憂?
4. 護士在你所提出的需要和擔憂後怎樣回應? 你是否滿意他們對你的回應?
5. 是甚麼原因使你向護士表達你的需要和擔憂?
6. 是甚麼原因使你不向護士表達你的需要和擔憂?
